# Micro-Foamed-Based Viscosity Reduction of SBS-Modified Asphalt and Its Physical and Rheological Properties

**DOI:** 10.3390/polym18060710

**Published:** 2026-03-14

**Authors:** Peifeng Cheng, Aoting Cheng, Yiming Li, Rui Ma, Youjie Chen

**Affiliations:** School of Civil Engineering and Transportation, Northeast Forestry University, Harbin 150040, China; chengaoting@nefu.edu.cn (A.C.); mrui@nefu.edu.cn (R.M.); chenyoujie@nefu.edu.cn (Y.C.)

**Keywords:** SBS-modified asphalt, micro-foamed process, crystalline hydrates, rheological properties, morphological evolution, asphalt viscosity reduction

## Abstract

Foaming technology can effectively reduce the viscosity of polymer-modified asphalt and significantly decrease energy consumption during pavement construction, making it an effective approach for achieving low-carbon pavement construction and maintenance. However, mechanically foamed asphalt relies on specialized equipment and requires strict parameter control. Although water-based foaming methods using zeolites or ethanol can alleviate these issues to some extent, they still present disadvantages such as significant variability in foaming performance and potential risks during transportation and construction. Therefore, this study investigates the feasibility of using crystalline hydrates with high water of crystallization for micro-foamed asphalt. Three types of micro-foamed SBS-modified asphalt (MFPA) were prepared using hydrates with different contents of water of crystallization. Physical property tests, foaming characteristic parameters, viscosity–temperature analysis, Fourier transform infrared spectroscopy (FTIR), adhesion tensile tests, scanning electron microscopy (SEM), and fluorescence microscopy were conducted to evaluate their effects on the physical and chemical properties, viscosity reduction performance, adhesion, and compatibility of SBS-modified asphalt. Furthermore, dynamic shear rheometer (DSR) tests, bending beam rheometer (BBR) tests, fatigue life modeling, and morphological analysis were employed to investigate the rheological properties, fatigue life, and bubble evolution behavior of the MFPA system. The results indicate that utilizing the thermal decomposition characteristics of crystalline hydrates with high water of crystallization (Na_2_SO_4_·10H_2_O, Na_2_HPO_4_·12H_2_O, and Na_2_CO_3_·10H_2_O) to release H_2_O and CO_2_ in SBS-modified asphalt for micro-foaming is a short-term reversible physical viscosity reduction process. The maximum expansion ratio (*ERmax*) of MFPA reaches 8–10, the half-life (*HL*) remains stable at approximately 180 s, and the foaming index (*FI*) peak is about 1160. The construction temperature can be reduced by 10–15%, and the viscosity reduction effect remains stable within 60 min. Compared with unfoamed SBS-modified asphalt, the compatibility, rutting resistance, and fatigue life of MFPA increase by approximately 65%, 32%, and 30%, respectively, while the low-temperature performance decreases by 18%. Under the same short-term and long-term aging conditions, MFPA exhibits better aging resistance. Specifically, its rutting resistance increases by 37%, and fatigue resistance improves by 30% compared with aged SBS-modified asphalt, while the low-temperature performance remains essentially unchanged.

## 1. Introduction

Asphalt pavements exhibit excellent performance and account for over 90% of high-grade roadways in China [1]. Among these, styrene-butadiene-styrene (SBS) modified asphalt is widely used in pavement engineering due to its superior service performance and durability [2]. However, the inherently high viscosity of SBS-modified asphalt at equivalent temperatures necessitates elevated mixing, paving, and compaction temperatures, which not only increases emissions of greenhouse gases and harmful fumes [2,3], exacerbating environmental pollution, but also poses serious health risks to construction personnel [4]. Therefore, warm-mix asphalt (WMA) technologies, which can reduce construction temperatures by 20–30 °C while maintaining satisfactory pavement performance [5,6], have become one of the most important solutions in global road construction over the past decade. According to the Asphalt Pavement Industry Survey on Recycled Materials and Warm-Mix Asphalt Usage: 2023 jointly issued by the U.S. Federal Highway Administration (FHWA) and the National Asphalt Pavement Association- (NAPA), the total asphalt production in the United States in 2023 was approximately 409 million tons, of which WMA accounted for 177 million tons, representing 43.3% of the total. Among the various WMA technologies, foamed techniques dominate, accounting for approximately 76.5% of total WMA production, which represents an increase of about 14% compared to 2018 [7].

At present, foaming technology for SBS-modified asphalt has demonstrated significant engineering advantages and environmental benefits [8,9]. Studies by Hu, Lu, and co-workers [10,11] have shown that this technology can reduce the production temperature of SBS-modified asphalt by approximately 20 °C, while decreasing energy consumption and carbon dioxide emissions by about 9.8% and 10%, respectively. He, Zhang, and co-workers [12,13] further reported that, under a low foaming water content (less than 2.0%), foamed SBS-modified asphalt exhibited approximately 1.2 times longer fatigue life and about a 24% improvement in rutting resistance compared with hot-mix asphalt (HMA), along with enhanced workability during construction. Lei, Tao, and co-workers [14,15] also demonstrated that the foaming process effectively reduced the construction temperature of SBS-modified asphalt pavements, and the resulting pavements exhibited superior riding quality, skid resistance, and rutting resistance compared with HMA. Nevertheless, this technology still presents certain limitations. Ma and co-workers [16,17] pointed out that the high-temperature and high-pressure conditions involved in conventional mechanical foaming may accelerate asphalt aging and performance deterioration. Liang and co-workers [18,19] found that residual moisture from mechanical foaming can form weak zones within the asphalt, thereby impairing its low-temperature performance and mechanical strength, and weakening the asphalt–aggregate interfacial bonding [20]. Meanwhile, Hu and co-workers emphasized that parameters such as foaming equipment type, gas flow rate, temperature, and pressure have a decisive influence on foaming performance. Differences between laboratory and field foaming equipment may result in discrepancies between the expected and actual performance of foamed asphalt in practical engineering applications [21,22].

Therefore, micro-foamed technologies characterized by mild release of material composition [23], simple preparation procedures, and the ability to achieve continuous and stable foamed over a wide temperature range (100–160 °C) have attracted increasing attention in recent years [24,25]. However, current studies on micro-foamed SBS-modified asphalt using zeolite or volatile substances as foamed agents still exhibit certain limitations. For instance, the production of synthetic zeolites requires specialized equipment and specific processing conditions, is sensitive to environmental factors, and exhibits significant performance variations among different types [26]. In addition, some synthetic zeolites may contain volatile organic compounds (VOCs) and other hazardous components, which can volatilize during construction [27]. Natural zeolites, on the other hand, present difficulties in standardization due to their complex composition and unstable moisture content [28]. Highly volatile substances such as ethanol are associated with potential risks during storage, transportation, and foamed processes [29], thereby limiting their range of application.

In contrast, the three inorganic crystalline hydrates Na_2_SO_4_·10H_2_O, Na_2_HPO_4_·12H_2_O, and Na_2_CO_3_·10H_2_O possess high and fixed crystalline-water contents (55.9–63.0%), contain no VOCs, are widely available, and exhibit stable thermochemical behavior. These characteristics make them promise and environmentally friendly micro-foaming agents with more controllable water-release properties than zeolite- or ethanol-based systems. Their predictable dehydration behavior enables the formation of stable micro-foamed structures without the safety concerns or standardization challenges associated with conventional foaming agents. When incorporated into SBS-modified asphalt through mechanical shearing and mixing, these hydrates can produce a short-term stable micro-foamed polymer system with improved foaming reliability, thereby reducing construction temperatures while mitigating the limitations observed in earlier micro-foaming approaches.

In summary, this study systematically investigated the foaming behavior of three crystalline hydrates with high water-of-crystallization content in SBS-modified asphalt and their influence on asphalt performance. Initially, physical property tests, foamed characteristic measurements, viscosity–temperature analysis, Fourier transform infrared spectroscopy (FTIR), adhesion tensile tests, scanning electron microscopy (SEM), and fluorescence microscopy (FM) were employed to assess the effects of crystalline hydrates on the physical and chemical properties, viscosity reduction efficiency, adhesion, and compatibility of SBS-modified asphalt. Subsequently, dynamic shear rheometer (DSR) and bending beam rheometer (BBR) tests, fatigue life modeling, and morphological analyses were conducted to evaluate the short- and long-term rheological behavior at high and low temperatures, fatigue performance, and bubble evolution characteristics of the micro-foamed asphalt systems. Finally, the mechanism underlying crystalline hydrate-induced micro-foaming was elucidated.

## 2. Materials and Methods

### 2.1. Materials

**Figure 1:**
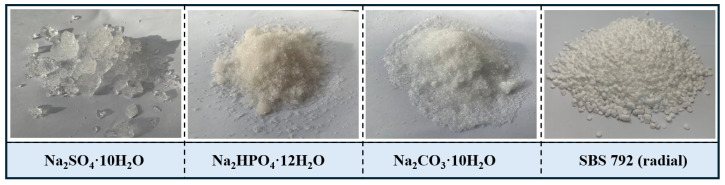
Micro-foamed materials and SBS modifier.

AH-90 matrix asphalt sourced from Panjin, Liaoning Province, and SBS 792 (radial structure) modifier supplied by (Beijing, China) Sinopec were used in this study. Their basic properties are presented in Table 1 and Table 2. The micro-foamed materials, Na_2_SO_4_·10H_2_O, Na_2_HPO_4_·12H_2_O, and Na_2_CO_3_·10H_2_O, were provided by Tianjin Tianli Chemical Co., Ltd. (Tianjin, Chian), all analytical grade, as shown in Figure 1.

### 2.2. Preparation

#### 2.2.1. Preparation of Micro-Foamed Asphalt

**Figure 2:**
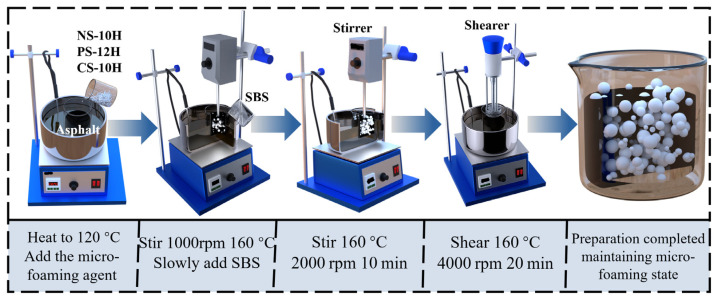
Preparation process.

AH-90 matrix asphalt was first heated to a fluid state at 120 °C. Subsequently, different dosages (1.0%, 3.0%, 5.0%, and 7.0%) of the micro-foamed materials—Na_2_SO_4_·10H_2_O, Na_2_HPO_4_·12H_2_O, or Na_2_CO_3_·10H_2_O—were added. The blend was initially stirred at 1000 rpm and 160 °C to gradually induce the micro-foamed effect, followed by the slow incorporation of 3.0 wt% SBS. The stirring speed was subsequently increased to 2000 rpm and maintained for 10 min. Thereafter, high-speed shearing was performed using a high-speed emulsification shearing machine at 4000 rpm and 160 °C for 20 min. The resulting binders were denoted as Na_2_SO_4_·10H_2_O/SBS (NS-10H), Na_2_HPO_4_·12H_2_O/SBS (PS-12H), and Na_2_CO_3_·10H_2_O/SBS (CS-10H) modified asphalts, along with a control SBS-modified asphalt prepared without micro-foamed materials. The preparation procedure is illustrated in Figure 2.

#### 2.2.2. Aging of Micro-Foamed Asphalt

Rolling Thin-Film Oven Test (RTFOT): In accordance with ASTM D6521 [35], the hot air flow was controlled at 4000 ± 200 mL/min, with a rotation speed of 15 rpm. The samples were aged at 163 ± 0.5 °C for 85 min to simulate the aging of asphalt during transportation, mixing, and paving processes.

Pressure Aging Vessel (PAV) Test: According to ASTM D2872 [36], asphalt samples obtained from the RTFOT were subjected to aging at a pressure of 2.1 MPa and 100 °C for 20 h to simulate the thermo-oxidative aging of asphalt pavements.

### 2.3. Methods

#### 2.3.1. Physical Property Test

In accordance with ASTM D5, ASTM D36, and ASTM D113, the penetration at 25 °C, softening point, and ductility at 5 °C were determined respectively, to evaluate the physical properties of micro-foamed asphalt with different types and dosages.

#### 2.3.2. Foamed Performance Test

The expansion ratio of MFPA at any given time, *ER*(*t*), was calculated using Equation (1). The *ER*(*t*)–time curve was smoothed according to Equation (2). The half-life (*HL*) was defined as the time required for the maximum expansion volume to decrease to 50%. The foamed index (*FI*), calculated using Equation (3), was determined as the area under the *ER*(*t*) curve within the *HL* period. Furthermore, the *ER*(*t*)–time relationship was simplified and fitted using Equation (4) to obtain the bubble collapse rate (*k*).

(1) $$ER(t)=\frac{h_{f}(t)-{h_{u}}_{f}}{{h_{u}}_{f}}$$

(2) $$ER(t)=1+ae^{-bt}+(ER_{\max}-a-1)e^{-ct}$$

(3) $$FI=\int_{t=0}^{t=HL\sec}ER(t)dt$$

(4) $$ER(t)=1+he^{-kt}$$

Here, the maximum expansion ratio (*ER_max_*) is defined as the ratio of the maximum expanded volume to the unfoamed volume of asphalt. $h_{f}(t)$ and ${h_{u}}_{f}$ represent the asphalt height at time t and the initial height before foamed, respectively. The parameters *a*, *b*, *c*, *h* and *k* fit the coefficients.

#### 2.3.3. Viscosity Test

Viscosity–Temperature Curves: Referring to ASTM D6925 [37] and AASHTO T312 [38], the Brookfield rotational viscosity test was adopted as the standard method to evaluate the viscosity reduction of asphalt. A No. 21 spindle was used at a rotational speed of 100 rpm, with the torque controlled within 10–98%. The viscosity of MFPA was measured over the temperature range of 105–180 °C at 15 °C intervals. At each temperature, readings were recorded every 60 s, and the average of three consecutive measurements was reported.

Viscosity Reduction Durability: The prepared micro-foamed asphalt was placed in a constant-temperature oven at 135 ± 1 °C. The rotational viscosity at 135 °C was measured at 20 min intervals. Based on the distribution pattern of the experimental data, the Boltzmann model was employed for curve fitting and the viscosity change curve of asphalt was drawn. The model equation is presented in Equation (5).

(5) $$V=A_{2}+\frac{A_{1}-A_{2}}{1+e^{\frac{t-t_{0}}{dt}}}$$

Here, A_1_ and A_2_ represent the upper and lower limits of the model respectively; t_0_ denotes the inflection point at the center of the curve; t is the storage time; and *dt* is the slope factor.

#### 2.3.4. FTIR Spectroscopy

Fourier transform infrared spectroscopy (FTIR) was conducted using a Nicolet iS50 spectrometer from Thermofisher Scientific (Waltham, MA, USA) to investigate the influence of micro-foamed materials on the characteristic functional groups of asphalt. The scanning range was 4000–500 cm^−1^, with 32 scans collected at a resolution of 4 cm^−1^.

#### 2.3.5. Compatibility Test

FM: To evaluate the compatibility of the micro-foamed polymer-modified asphalt system, fluorescence microscopy (ZEISS 301) was employed to observe the microstructure of asphalt prepared using different processing methods at 100× magnification. The samples were prepared using the hot-drop method, in which the micro-foamed asphalt was dropped onto a glass slide maintained at 110 °C, followed by gently placing a cover slip over the material. The temperature was maintained at 110 °C until the asphalt surface became naturally smooth. The FM images obtained were processed using MATLAB R2024a to calculate the coefficient of variation (CV) and Shannon entropy (SE) [39]. The corresponding calculation formulas are presented in Equations (6) and (7).

(6) $$CV=\frac{\sigma }{\mu }$$

Here, σ represents the local standard deviation, μ denotes the local mean value.

(7) $$SE=-\sum_{i=1}^{L}P_{i}\log_{2}(P_{i})$$

Here, $P_{i}$ is the probability of pixels with a gray level of i, and L is the total number of gray levels.

Scanning Electron Microscopy (SEM): The surface morphology of micro-foamed asphalt was examined using a Hitachi Regulus8100 scanning electron microscope from Hitachi High-Tech Corporation (Tokyo, Japan). The operating voltage was set at 20 kV, and the magnifications were 0.5 k×, 1.0 k×, 5.0 k×, and 10.0 k×.

#### 2.3.6. Adhesion Tensile Test

**Figure 3:**
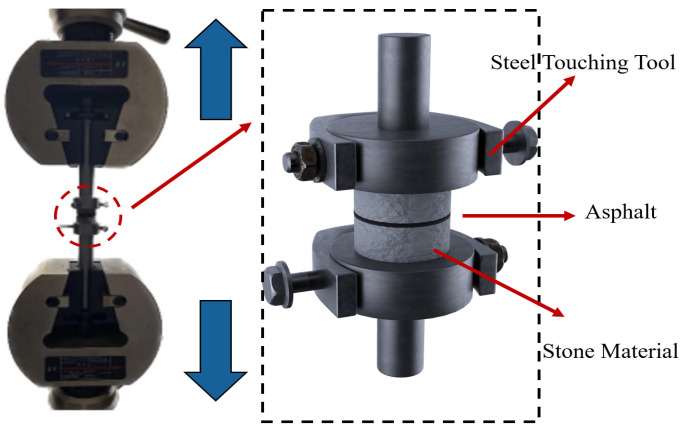
Schematic illustration of the tensile test.

Adequate adhesion is essential to ensure the pavement performance of MFPA. A tensile test was conducted using a universal testing machine to evaluate the adhesion capacity of free-water-foamed and crystalline-water-based micro-foamed asphalt to aggregates. In the test, 5 g of asphalt was applied to the surfaces of two cylindrical aggregate specimens (25 mm in diameter and 15 mm in thickness). The specimens were pressed and controlled the asphalt film thickness at 10 ± 1 μm. After cooling at room temperature, the bonded specimens were conditioned in a 5 °C water bath for at least 2 h. Subsequently, the samples were mounted onto a direct tensile fixture and fixed to a universal testing machine. The loading rate was set at 10 mm/min to induce direct tensile failure at the aggregate–asphalt interface. Three parallel tests were conducted for each group. A schematic diagram of the tensile test setup is shown in Figure 3.

#### 2.3.7. Resistance to Rutting Dissipation Work

A dynamic shear rheometer MCR 301 was used with a controlled strain of 0.1%. The testing frequencies were 0.01, 0.1, 1, 5, and 10 Hz. The rutting resistance was evaluated over the temperature range of 46–82 °C at 12 °C intervals. The high-temperature rutting resistance of asphalt was characterized using the work dissipated for rutting potential. Under constant strain conditions, a higher resistance to rutting dissipation work per loading cycle indicates stronger rutting resistance. The calculation was based on Equation (8).

(8) $$W_{C}=\pi {\sigma_{0}}^{2}/(G^{*}/\sin\delta )$$

Here, W_C_ represents the resistance to rutting dissipation work per loading cycle, G*/sinδ is the rutting factor, σ_0_ is the applied stress during each loading cycle.

#### 2.3.8. BBR Test

According to ASTM D6648 [40], a bending beam rheometer (BBR) TE-BBR SD was employed to evaluate the low-temperature performance of micro-foamed asphalt with different storage durations and aging levels. The test temperatures were −12 °C, −18 °C, and −24 °C, with three parallel tests conducted at each temperature. Combined with current research, the k value, defined as the ratio of stiffness modulus (S) to creep rate (m) [41], was adopted as the evaluation index of low-temperature performance. A higher k value indicates poorer low-temperature performance. The calculation formula is given as follows:

(9) $$k=\frac{S}{m}$$

#### 2.3.9. Fatigue Life Prediction Model

A fatigue life prediction model was established based on frequency sweep and linear amplitude sweep (LAS) tests. The frequency sweep was conducted at 25 °C over a frequency range of 0.2–30 Hz with a strain level of 0.1%. The LAS test was performed at 25 °C with an amplitude scanning range of 0.1–30%, a loading frequency of 10 Hz, and a total testing duration of 310 s. The model construction method is as follows [42,43].

(10) $$\log G^{\prime }=m\log\omega +b$$

(11) $$\alpha =\frac{1}{m}$$

Here, $G^{\prime }$ represents the storage modulus, ω denotes the angular velocity, m is the slope of the fitted linear relationship between the storage modulus and the logarithmic frequency, and b is the intercept.

(12) $$D(t)≅{\sum_{i=1}^{N}\left[\pi {\gamma_{0}}^{2}(G^{*}\sin\delta_{i-1}-G^{*}\sin\delta_{i-1})\right]}^{\frac{\alpha }{1+\alpha }}{\left(t_{i}-t_{i-1}\right)}^{\frac{\alpha }{1+\alpha }}$$

Here, D(t) denotes the damage at time t, $t_{i}$ represents the i-th testing time, *N* is the total number of loading cycles, and $\gamma_{0}$ is the applied strain.

(13) $$C_{\left(t\right)}=C_{0}-C_{1}{\left(D_{\left(t\right)}\right)}^{C_{2}}$$

(14) $$C_{\left(t\right)}=\frac{{G^{*}}_{\left(t\right)}}{{G^{*}}_{initial}}$$

Here, $C_{0}$ is the initial value of C, specified as 1, while $C_{1}$ and $C_{2}$ are fitting coefficients derived from power-law linearization.

(15) $$D_{f}={\left(\frac{C_{0}-C_{P}}{C_{0}}\right)}^{\frac{1}{C_{2}}}$$

(16) $$k=1+(1-C_{2})\alpha$$

(17) $$N_{f}=\frac{f{\left(D_{f}\right)}^{k}}{k{\left(\pi C_{1}C_{2}\right)}^{\alpha }}{\left(\gamma_{\max}\right)}^{-2\alpha }$$

Here, $D_{f}$ is the cumulative damage parameter, $C_{P}$ represents the peak stress, $N_{f}$ denotes the fatigue life, and $\gamma_{\max}$ is the maximum applied strain, f = 10 Hz.

#### 2.3.10. Morphological Evolution

The prepared micro-foamed asphalt was subjected to thermal storage in a constant-temperature oil bath at 135 ± 1 °C for 2.5 h. The following procedures were repeated at 0.5 h intervals:

(1) Macroscopic observation: A fixed shooting device was positioned 20 cm directly above the asphalt surface to shoot the bubble state of the upper surface at a constant angle. The acquired images were processed using MATLAB with regional segmentation to determine the foam density and bubble size distribution.
(2) Microscopic observation: The hot-drop method was employed by dropping the asphalt onto a glass slide preheated to 135 °C, followed by gently placing a cover slip on top. The specimen was immediately frozen using liquid nitrogen and subsequently stored in a −20 °C freezer for at least 3 h. The microstructural morphology was then observed using a BKM-X500 optical microscope from Chongqing Optec Instrument Co., Ltd. (Chongqing, China) at a constant magnification of 20× to analyze the evolution of microscopic morphology.

## 3. Results and Discussion

### 3.1. Physical Properties

**Figure 4:**
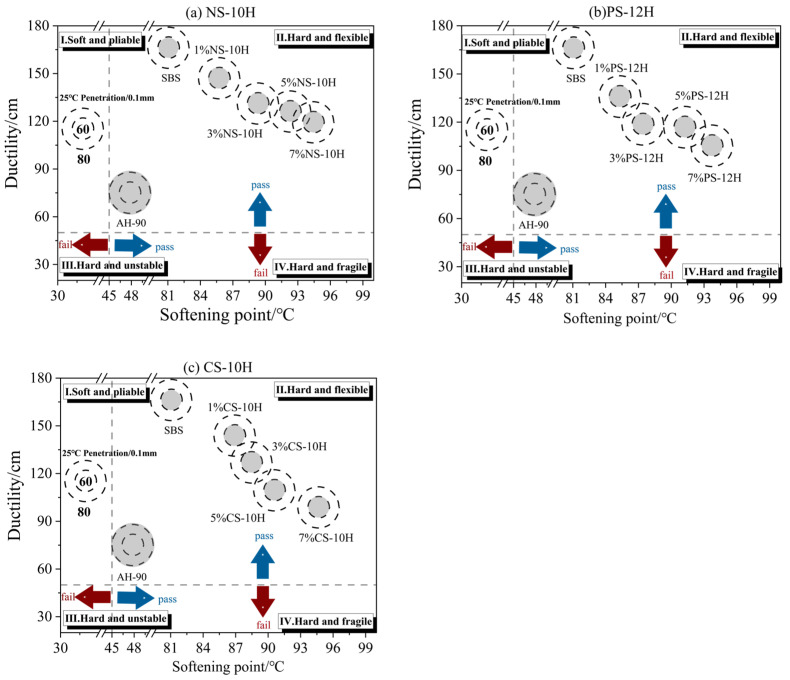
Basic physical properties of micro-foamed SBS-modified asphalt.

The results of the physical property tests for MFPA are presented in Figure 4. Overall, the incorporation of SBS significantly enhanced the elasticity and deformation resistance of asphalt. Compared with the matrix asphalt, the softening point and ductility of the modified asphalt increased by 76% and 123%, respectively. The incorporation of inorganic crystalline hydrates influences the performance of asphalt, and this effect becomes more pronounced with increasing dosage. Compared with SBS-modified asphalt, the softening point of MFPA increased by 6%, while its ductility decreased by 12%. This behavior is mainly attributed to the continuous release of water vapor from the micro-foamed asphalt under high-temperature conditions, which introduces many micropores into the asphalt matrix. These microporous structures reduce the continuity of the SBS elastic network [44,45], making it more susceptible to localized stress concentration during tensile deformation. Consequently, a reduction in ductility is observed. Meanwhile, the inorganic particles generated from the decomposition of the micro-foamed materials can physically interact with the SBS phase and, together with the microporous structure, synergistically enhance the overall stiffness of the system. At ambient temperature, the influence of the microporous structure on the local penetration resistance of the needle is relatively weak, and the SBS network remains stable; therefore, the micro-foamed process has a limited effect on the penetration of polymer-modified asphalt. Overall, MFPA exhibits satisfactory comprehensive performance and meets the requirements of relevant specifications.

### 3.2. Foamed Characteristics

**Figure 5:**
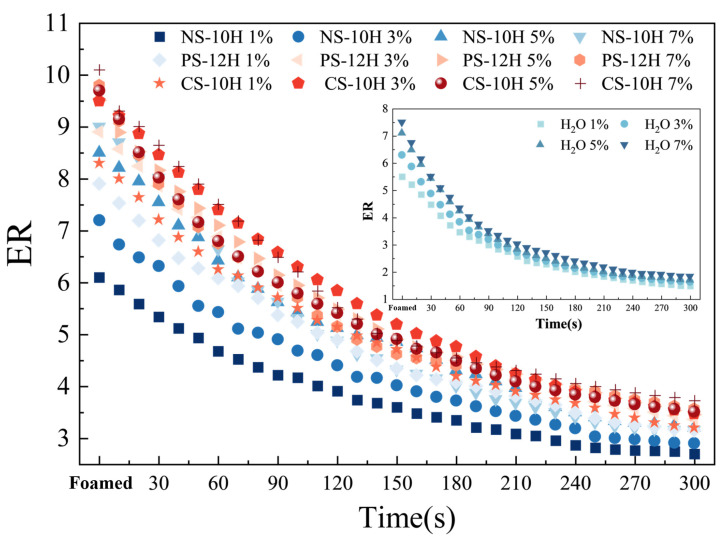
ER–t curves of MFPA at different dosages.

*ER_max_* and *HL* are key parameters for evaluating the volumetric expansion behavior and foamed stability of micro-foamed asphalt. A higher *ER_max_* indicates a more pronounced volumetric expansion during the foamed process and a more significant viscosity reduction effect [44]. Moreover, a larger *HL* value suggests a longer duration of the viscosity reduction effect. By measuring the expansion ratio of MFPA at different time intervals from the onset of foamed to 300 s after foamed, the *ER*(*t*) curves were obtained, as shown in Figure 5. The ER values of MFPA exhibit an exponential decay trend with increasing time. Compared with water-foamed asphalt, the *ER_max_* of MFPA increased by approximately 25%, and the *HL* was extended by about 30%. Previous studies have generally suggested that, to ensure satisfactory pavement performance of foamed asphalt, the minimum *ER_max_* should exceed 8, and the half-life should be longer than 20 s. The results indicate that when the dosage of micro-foamed materials reaches 3% or higher, MFPA meets the relevant specification requirements and can provide an effective viscosity reduction.

**Figure 6:**
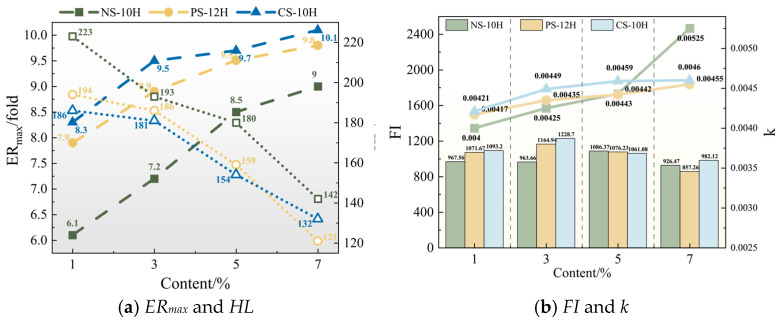
Foamed characteristic parameters.

The foaming characteristic parameters are presented in Figure 6a. At the same dosage, the micro-foaming capability follows the order: CS-10 > PS-12H > NS-10H. These differences are mainly attributed to the variation in crystalline water content among the three materials, which are 62.9%, 60.4%, and 55.9%, respectively. In addition, the decomposition of CS-10 is accompanied by the release of CO_2_, which provides an additional gas source and synergistically enhances the viscosity reduction effect. For the same type of MFPA, *ER_max_* gradually increases with increasing dosage, whereas *HL* shows a pronounced decreasing trend. This indicates that the viscosity reduction effect is enhanced, while its duration becomes shorter. This behavior is mainly attributed to the fact that the micro-foaming process of MFPA is driven by the rapid release of crystalline water at elevated temperatures; higher dosages lead to more intense vaporization of crystalline water. The concentrated release of water vapor results in a higher *ER_max_* of the asphalt; however, the reduction in surface energy in the large-bubble foam system accelerates structural collapse, leading to a significant shortening of *HL*.

However, *ER_max_* and *HL* are based solely on local features of the *ER*(*t*) curve and cannot fully represent the overall micro-foamed intensity of asphalt. Therefore, the FI and k values were introduced to quantify the comprehensive foamed performance of MFPA. As shown in Figure 6a, the k value represents the decay rate of the *ER*(*t*) curve; a smaller k value indicates higher system stability. The FI value corresponds to the area under the ER curve within *HL* and is used to characterize the overall foamed intensity. As shown in Figure 6b, the *k* value of MFPA gradually increases with the increasing dosage of inorganic crystalline hydrates. Specifically, for NS-10H, increasing the dosage from 5% to 7% results in a 19% increase in *k*, indicating that excessive addition of micro-foamed agents may lead to unstable foamed performance and that the dosage should not be excessively high. The FI value of MFPA exhibits a trend of first increasing and then decreasing with increasing dosage. Among them, the maximum FI values for NS-10H, PS-12H, and CS-10H are 1086.37, 1164.94, and 1228.70, reached at dosages of 5%, 3%, and 3%, respectively. Beyond these dosages, the rapid collapse of MFPA bubbles dominates over the increase in expansion ratio, resulting in a decrease in the overall foamed intensity.

In practical pavement construction, the foaming characteristics of MFPA directly support mixture workability and temperature control. *ERmax* values of 8–10 and *HL* values above 180 s indicate that MFPA sustains adequate expansion and viscosity reduction throughout typical plant mixing and compaction periods, without requiring specialized foaming equipment. Additionally, the predictable release of crystalline water enables stable foaming over a wide temperature range, allowing MFPA to be integrated into existing asphalt plants through conventional dry mixing. These characteristics collectively demonstrate strong feasibility for field implementation and temperature reduction during construction.

### 3.3. Viscosity–Temperature Curves and Viscosity Reduction Durability 

**Figure 7:**
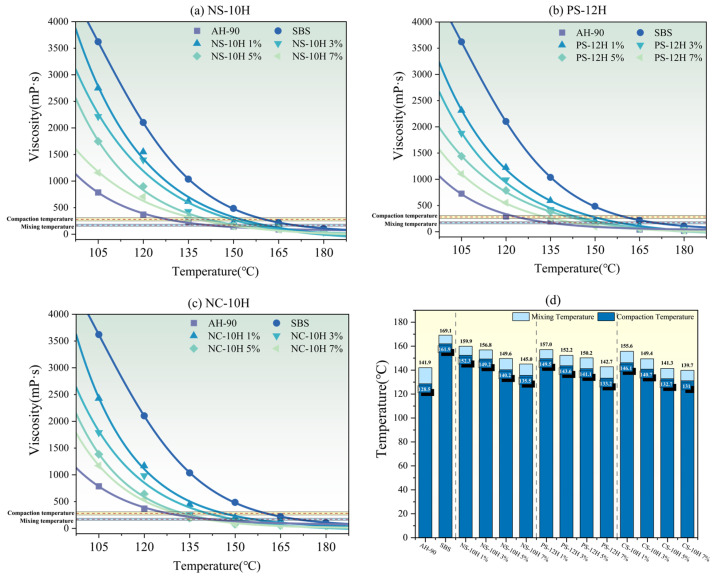
(**a**–**c**) Viscosity–temperature curves; (**d**) Compaction and mixing temperatures.

According to ASTM D 6925 and AASHTO T 312, the temperature range corresponding to a viscosity of 170 ± 20 mPa·s is defined as the mixing temperature range of asphalt, while the temperature range corresponding to a viscosity of 280 ± 30 mPa·s is defined as the compaction temperature range [46,47]. The viscosity–temperature curves of MFPA with different types and dosages are shown in Figure 7a–c. As can be observed from the figures, the micro-foamed process can significantly reduce the viscosity of polymer-modified asphalt. At the same temperature, the viscosity of MFPA continuously decreases with increasing dosage of micro-foamed materials. Meanwhile, the viscosity of MFPA is consistently lower than that of unfoamed SBS-modified asphalt but higher than that of AH-90 base asphalt. Based on the specifications, the compaction and mixing temperatures of each asphalt were determined, as shown in Figure 7d. The compaction and mixing temperatures of SBS-modified asphalt are approximately 161.9 °C and 169.1 °C, respectively. Compared with SBS-modified asphalt, the average compaction temperatures of NS-10H, PS-12H, and CS-10H decrease by approximately 11%, 12%, and 15%, respectively, while the corresponding mixing temperatures are reduced by about 10%, 11%, and 13%. Compared with SBS-modified asphalt, the average compaction temperatures of NS-10H, PS-12H, and CS-10H decrease by approximately 11%, 12%, and 15%, respectively, while the corresponding mixing temperatures are reduced by about 10%, 11%, and 13%. Overall, the viscosity reduction effects of the three MFPA systems are comparable. The observed temperature differences among NS-10H, PS-12H, and CS-10H mainly arise from their distinct thermophysical behaviors during heating. The three hydrated salts differ in crystalline water content and dehydration onset temperatures, which directly affect the amount and timing of micro-bubble generation in asphalt. PS-12H contains more crystalline water and dehydrates earlier, producing relatively stronger foaming at lower temperatures. In contrast, CS-10H undergoes both dehydration and partial thermal decomposition, releasing additional CO_2_ that enhances the foaming effect at higher temperatures and leads to the lowest compaction temperature. These variations in water release and gas-generation mechanisms ultimately result in the different mixing and compaction temperatures observed among the three MFPA systems.

**Figure 8:**
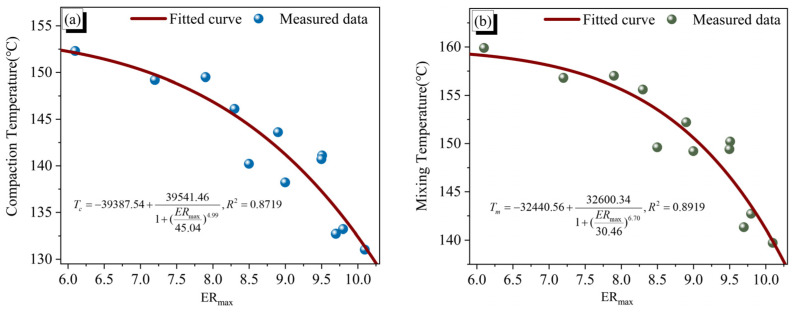
(**a**) Correlation between compaction temperature and *ERmax*; (**b**) Correlation between mixing temperature and *ERmax*

To enable a rapid evaluation of the viscosity reduction efficiency induced by the micro-foamed process, this study established a correlation model between *ER_max_* of MFPA and the compaction or mixing temperature. A generalized Sigmoid function was employed for model fitting, and the effects of compaction temperature and mixing temperature on the expansion ratio were comparatively analyzed. The mathematical form of the model is presented in Equation (18), and the corresponding fitting results are illustrated in Figure 8.

(18) $$T=A_{2}+\frac{A_{1}-A_{2}}{1+{\left(\frac{ER_{\max}}{B_{0}}\right)}^{p}}$$

In this model, T_c_ and T_m_ denote the compaction temperature and mixing temperature, respectively; A_1_, A_2_, B_0_ and p are fitting parameters.

As shown in Figure 8, both the mixing temperature and compaction temperature exhibit decreasing trends with increasing *ER_max_*. The fitted models yield R^2^ values exceeding 0.85, indicating a strong negative correlation. These results demonstrate that the *ER_max_* of MFPA can serve as an effective indicator for predicting the mixing and compaction temperatures during asphalt construction. Notably, the R^2^ value associated with the mixing temperature is slightly higher, suggesting that it is more sensitive to the volumetric expansion induced by the micro-foaming process.

**Figure 9:**
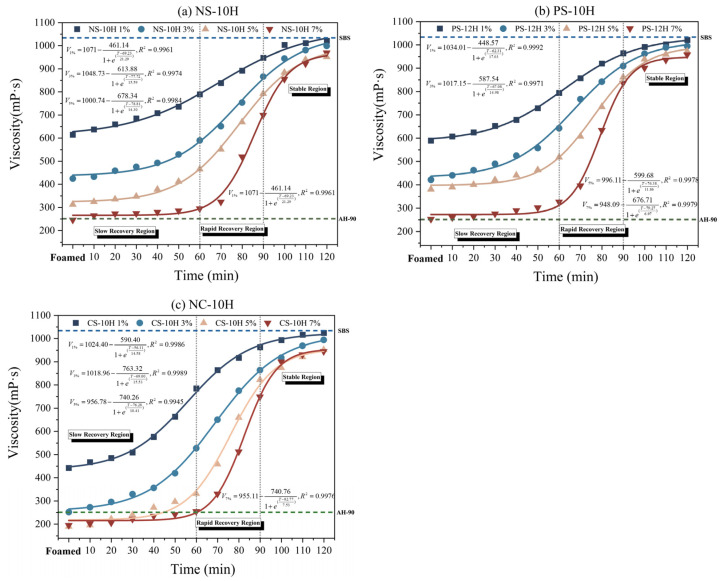
Viscosity recovery curves of MFPA.

Figure 9 presents the viscosity recovery curves of MFPA after foamed, which can be used to effectively evaluate the attenuation of the viscosity reduction effect during storage and transportation, as well as to predict the practical durability of the micro-foamed-based viscosity reduction. With increasing time, the viscosity of MFPA exhibits a three-stage evolution, characterized by an initial slow increase, followed by rapid recovery, and finally reaching a stable plateau, which overall conforms well to a Boltzmann-type evolution model. Variations in the dosage of the micro-foamed material significantly affect the initial viscosity of MFPA, whereas their influence on the long-term stabilized viscosity is negligible. With increasing MFPA dosage, the viscosity recovery rate during the intermediate stage gradually decreases. During the early stage of micro-foamed (0–60 min), crystallization water is dispersed in the asphalt in the form of dense and continuously generated bubbles, which enhances the mobility of the polymer-modified system and results in a slow increase in viscosity. As storage time increases into the intermediate stage (60–90 min), the bubble concentration gradually decreases, leading to an increase in the interfacial energy between phases. Consequently, intermolecular interactions among the asphalt components are re-established, and the viscosity enters a rapid recovery stage characterized by a steep upward trend. In the late stage of micro-foamed (90–120 min), the bubbles are almost completely dissipated, with negligible residual moisture remaining in the system, and the viscosity recovers to more than 95% of that of the non-foamed asphalt. After 12 h of storage, the viscosities of all MFPA types returned to their original levels, demonstrating that the micro-foaming process provides a short-term and reversible viscosity reduction effect. Taking viscosity at 60 min as a reference, CS-10H exhibits the best viscosity reduction stability, followed by NS-10H and PS-12H. The viscosity recovery ratios at 60 min are 46%, 52%, and 55%, respectively. These viscosity-recovery results correspond well to the foamed-stability indicators obtained from the expansion-ratio tests. A lower *HL* value reflects a shorter persistence of the foamed structure, meaning that bubbles dissipate more rapidly and the viscosity of asphalt recovers earlier and faster. Conversely, CS-10H exhibits the longest *HL* and highest overall foamed intensity, which slows the collapse of micro-bubbles and results in the lowest viscosity-recovery ratio at 60 min. Therefore, the ranking of viscosity reduction durability (CS-10H > NS-10H > PS-12H) matches the stability characteristics indicated by *HL* and FI, demonstrating strong consistency between the two evaluation methods.

In practical construction, the viscosity–temperature characteristics of MFPA directly determine its workability and energy-saving potential. The observed 10–15% reduction in mixing and compaction temperatures aligns well with typical production and paving conditions, indicating that MFPA can be incorporated into existing mixing plants without modifying equipment or altering workflow. Moreover, the predictable viscosity-recovery behavior ensures that the material maintains suitable workability during transport and laydown, supporting stable field operations and reducing thermal sensitivity under variable construction temperatures.

The slope factor *dt* in the viscosity recovery model characterizes the rate of viscosity recovery and can be regarded as a potential indicator for evaluating the viscosity reduction stability of MFPA. To verify the reliability of *dt* as a rapid evaluation indicator, a correlation analysis between *HL* and *dt* was conducted, and the corresponding correlation model is expressed in Equation (19).

(19) dt=a+kHL

Here, *a* and *k* are fitting parameters of the model.

**Figure 10:**
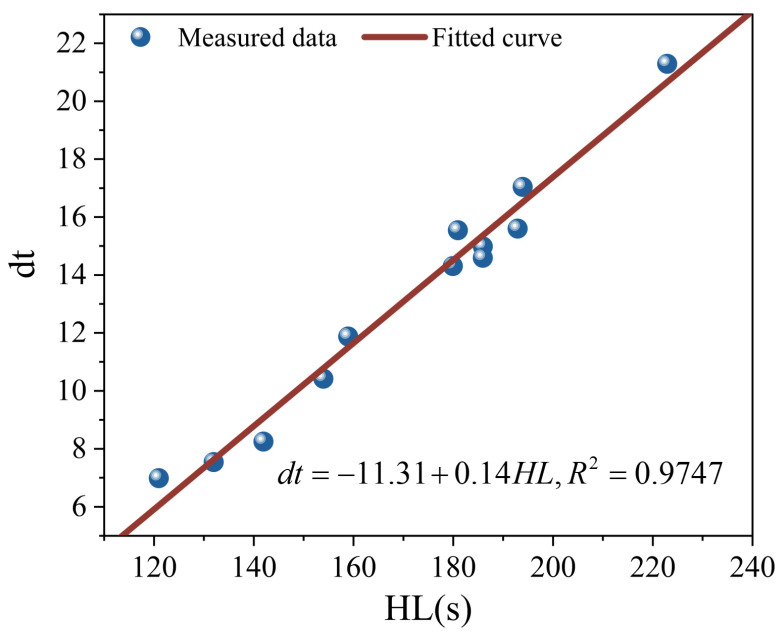
Correlation analysis between *HL* and *dt.*

As shown in Figure 10, a pronounced positive linear correlation is observed between *HL* and *dt*, with an R^2^ value exceeding 0.95, indicating a high fitting accuracy. These results demonstrate that both the foamed characteristic parameters and the viscosity recovery behavior at 135 °C can effectively evaluate the viscosity reduction stability of MFPA. In practical engineering applications, the viscosity reduction durability of MFPA can be rapidly predicted by simply determining *HL*.

### 3.4. Chemical Properties

**Figure 11:**
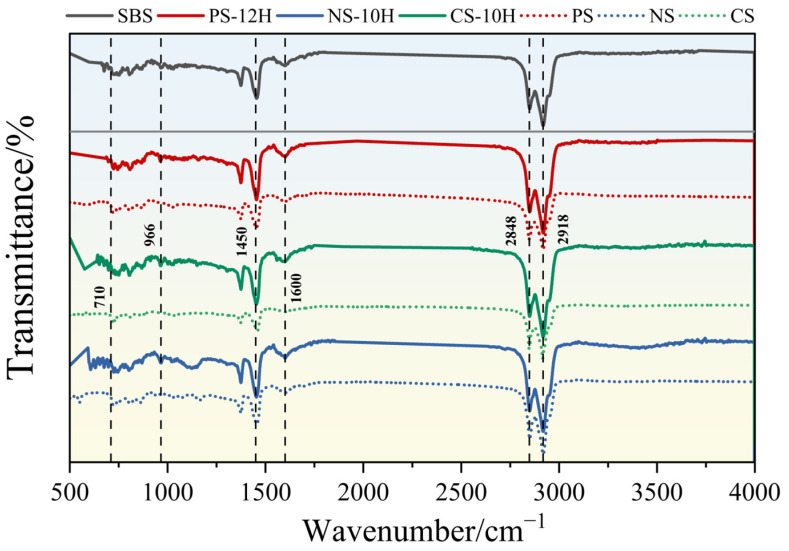
FTIR spectra.

FTIR is a commonly used technique for analyzing the chemical composition and functional group structures of materials and has been widely applied to evaluate chemical changes in asphalt before and after modification. The SBS-modified asphalt and the inorganic salt carrier without crystallization water were used as reference materials to comparatively analyze the chemical structural characteristics of MFPA, as shown in Figure 11.

Overall, the micro-foamed process does not alter the chemical functional group composition of the asphalt, indicating that the viscosity reduction effect is dominated by a typical physical process. As shown in Figure 11, the FTIR spectra of all seven asphalt samples exhibit characteristic C-H stretching vibrations of saturated alkyl groups at 2918 cm^−1^ and 2848 cm^−1^, along with aromatic C-C stretching vibrations in the range of 1600–1500 cm^−1^. The absorption bands at 966 cm^−1^ and 700–760 cm^−1^ are attributed to the 1,4-cis butadiene units and the out-of-plane bending vibrations of styrene units, respectively. All samples retain the typical spectral features of SBS-modified asphalt [48,49]. Compared with the SBS-modified asphalt, the characteristic peak intensities of the micro-foamed samples are generally enhanced, whereas the samples containing the inorganic salt carrier without crystallization water exhibit a noticeable decrease in peak intensity. This phenomenon is mainly attributed to the generation of numerous fine bubbles within the asphalt during the micro-foamed process, forming a porous structure analogous to a “honeycomb” or “sponge”, which increases infrared light scattering and the effective optical path length within the material, thereby enhancing the absorption intensity. In contrast, the anhydrous inorganic salt carrier exhibits negligible foamed behavior due to its limited gas release. Its particles mainly exist in the asphalt in the form of surface coverage or dispersion, exerting a dilution effect on the asphalt components and consequently weakening the absorption intensities of the intrinsic functional groups.

### 3.5. Compatibility Analysis

**Figure 12:**
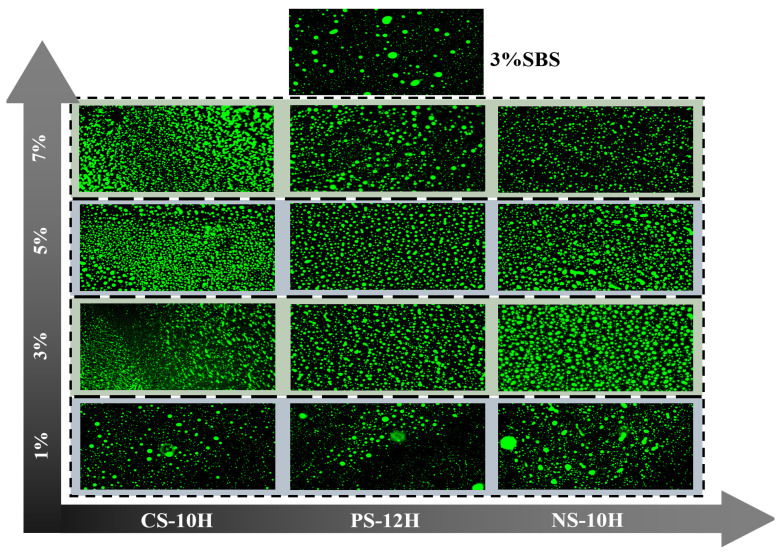
FM images of MFPA at different dosages.

Due to the significant differences in solubility and density between the SBS phase and the asphalt matrix, pronounced phase separation tends to occur during storage [50]. Fluorescence microscopy was employed to observe the spatial distribution of SBS within the asphalt and to evaluate the effect of the micro-foamed process on the compatibility of the polymer system, with the results shown in Figure 12.

As shown in Figure 12, the incorporation of micro-foamed materials significantly improves the compatibility of SBS-modified asphalt. Compared with the blocky agglomeration and irregular distribution of the SBS phase observed in non-foamed polymer-modified asphalt, the fluorescence domains in micro-foamed asphalt are finer and more uniformly dispersed. Furthermore, the dosage of the micro-foamed material has a pronounced effect on the compatibility of the polymer system, with both excessively high and excessively low dosages being unfavorable for the uniform distribution of the SBS phase. For all three types of micro-foamed asphalt, the fluorescence micrographs at dosages of 1% and 7% show similar spot-like large-scale agglomerations with pronounced variations in domain size, along with locally formed dark regions lacking SBS-phase distribution. In contrast, within the dosage range of 3–5%, the SBS phase exhibits a markedly improved dispersion state.

**Figure 13:**
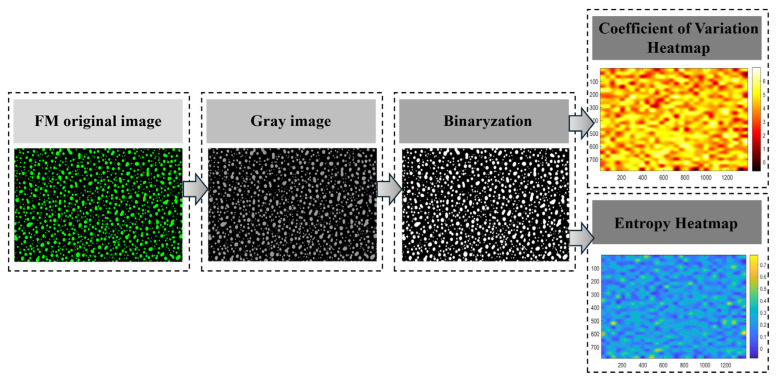
Image processing workflow for FM analysis.

To achieve an accurate quantitative comparison, FM images were processed using MATLAB (as illustrated in Figure 13), and the CV and SE were calculated according to Equations (6) and (7). Lower CV values and higher SE values indicate a higher degree of compatibility with the polymer system [39].

**Figure 14:**
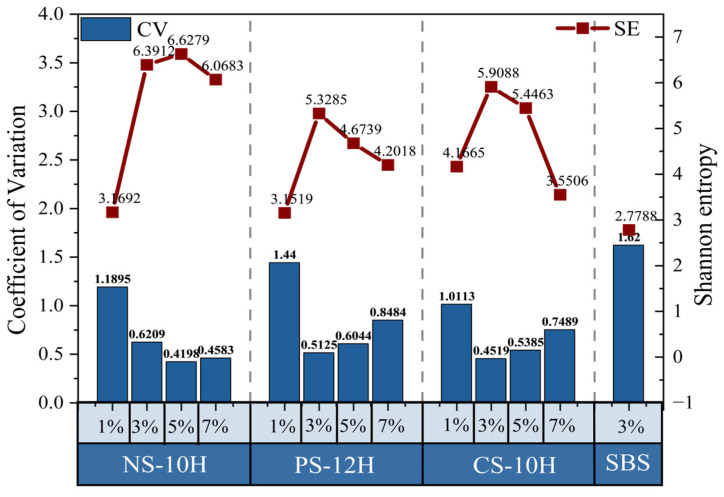
Dual-*Y*-axis plot of CV and SE.

The quantified CV and SE data were summarized and statistically analyzed, and the results are presented in Figure 14. Overall, compared with SBS-modified asphalt, the CV values of NS-10H, PS-12H, and CS-10H asphalts decreased by 59%, 47%, and 58%, respectively, while the corresponding SE values increased by 100%, 56%, and 72%. The combined trends of CV reduction and SE enhancement demonstrate that the micro-foaming process effectively improves the compatibility of polymer-modified asphalt. Furthermore, as the dosage of the micro-foamed material increases, the SE value exhibits an overall upward trend, indicating the disappearance of large bright or dark regions in the FM images, an expansion of grayscale distribution, and a progressive increase in image structural complexity. In contrast, the CV value shows a strong negative correlation with the micro-foamed material dosage and gradually decreases with increasing dosage. This trend reflects the evolution of FM image features from simple agglomeration toward a denser and more complex state, with finer textures and particulate features progressively emerging. However, when the dosage exceeds an optimal range, the compatibility of MFPA deteriorates. At low dosages, the number of generated bubbles is insufficient and difficult to stabilize, resulting in inadequate interfacial viscosity reduction, which allows SBS particles to re-agglomerate under the surface tension of the asphalt matrix. At excessively high dosages, large and densely distributed bubbles generated during the micro-foaming process tend to drive polymer particles toward bubble boundaries, causing localized accumulation. Meanwhile, the increased stress on bubble walls promotes bubble rupture, and excess water forms thin water films that further weaken the interfacial interaction between the polymer and the base asphalt. Based on the comprehensive analysis, NS-10H, PS-12H, and CS-10H asphalts exhibit the most pronounced improvement in compatibility at dosages of 5%, 3%, and 3%, respectively.

**Figure 15:**
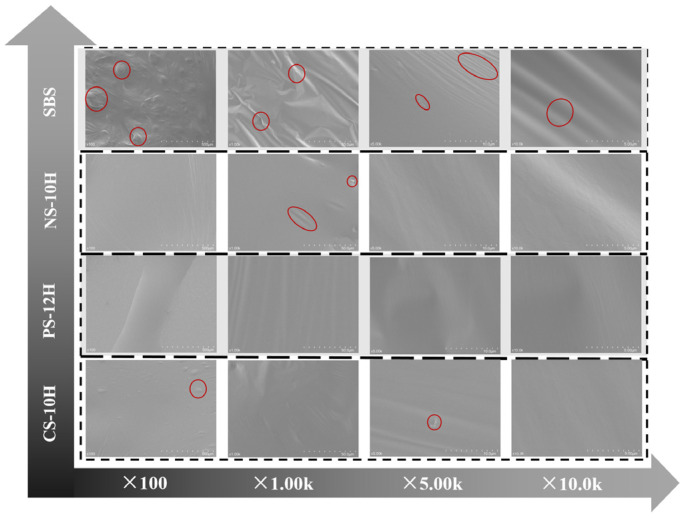
SEM images at different magnifications.

Based on the optimal dosages determined above (5% for NS-10H, 3% for PS-12H, and 3% for CS-10H), scanning electron microscopy (SEM) observations were further conducted on the micro-foamed polymer-modified asphalts at magnifications ranging from 100× to 10,000×. The compatibility of the micro-foamed system was validated from the perspectives of polymer phase distribution and interfacial morphology, as shown in Figure 15. It can be observed that the bubbles generated during the micro-foamed process are in a metastable state, and no residual crystallization water remains after solidification. During cooling and flow, the asphalt matrix refills the bubble cavities and eliminates microbubbles; therefore, no pore structures are observed on the SEM fracture surfaces. Meanwhile, compared with conventional SBS-modified asphalt, the fracture surfaces of the three MFPA samples appear smoother and cleaner, with finer textures and no pronounced particle protrusions, maintaining a flat and continuous morphology even at high magnifications. In contrast, the fracture surface of SBS-modified asphalt exhibits evident particle protrusions and locally rough regions, which manifest as irregular wrinkles and island-like agglomerations at high magnifications. These morphological differences indicate that the micro-foamed process optimizes the dispersion of polymers within the asphalt matrix, thereby effectively enhancing the overall compatibility of the system.

### 3.6. Adhesion Performance

**Figure 16:**
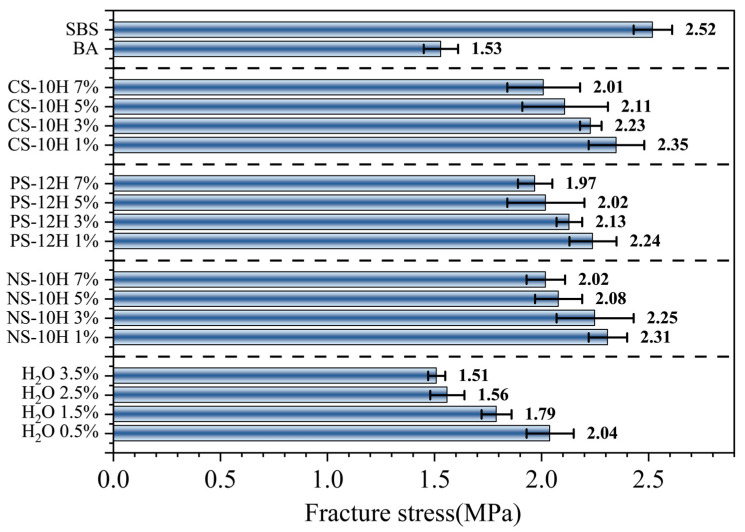
Adhesion fracture stress of asphalt.

The tensile test results of asphalt–aggregate specimens are presented in Figure 16. The adhesion performance followed the order: SBS-modified asphalt > CS-10 asphalt > PS-12H asphalt > NS-10H asphalt > free-water-foamed SBS-modified asphalt > matrix asphalt. It is evident that the incorporation of SBS significantly enhanced the adhesion between asphalt and aggregate, resulting in an approximately 65% increase compared with the matrix asphalt. However, the application of the free-water foamed process to SBS-modified asphalt led to an approximately 32% reduction in adhesion performance. In contrast, when Na_2_SO_4_·10H_2_O, Na_2_HPO_4_·12H_2_O, and Na_2_CO_3_·10H_2_O were introduced during the preparation of SBS-modified asphalt via the micro-foamed process, the adhesion performance decreased by only 14.1%, 17.1%, and 13.6%, respectively, representing an improvement of approximately 17% compared with the free-water foamed method. This difference is primarily attributed to the distinct foamed mechanisms. Free water foamed introduces non-uniform bubble structures into the polymer system, with a tendency for residual moisture to remain, which reduces the effective contact area between asphalt and aggregate and consequently weakens adhesion performance [51,52]. However, crystalline hydrates release water more stably and moderately upon heating, allowing for more complete dehydration and less residual moisture. As a result, under the same overall water content, MFPA exhibits a smaller loss in adhesion performance.

### 3.7. High-Temperature Rutting Resistance

**Figure 17:**
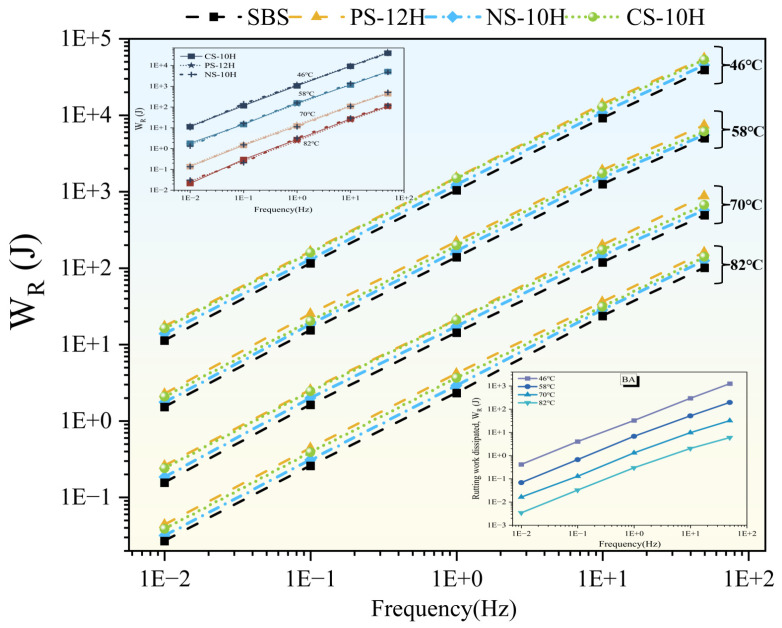
W_R_ at different temperatures and loading frequencies.

The rutting resistance of asphalt binders was evaluated using the work dissipated for rutting (W_R_), and the results are shown in Figure 17. Before and after the micro-foamed treatment, the variation trends of high-temperature performance were consistent. At a constant loading frequency, W_R_ gradually decreased with increasing temperature, whereas at a constant temperature, W_R_ increased with increasing frequency. Compared with the matrix asphalt, the W_R_ of SBS-modified asphalt increased by approximately 14–30 times. The micro-foamed process further provided an enhancement in the high-temperature rutting resistance of polymer-modified asphalt, resulting in an additional increase of about 30% in W_R_. In contrast, the inorganic carrier without crystalline water exhibited a negligible effect on the rutting resistance of asphalt. These phenomena can be attributed to the micro-foamed treatment, which effectively reduces asphalt viscosity and improves the compatibility of the polymer phase in SBS-modified asphalt. Consequently, polymer dispersion in MFPA is enhanced, leading to the formation of a more stable three-dimensional polymer network. In addition, the fine and enclosed bubbles formed within the SBS network create an internal spatial support system, which provides elastic buffering and stress dissipation under cyclic loading. These findings are consistent with those reported in recent related studies [19,53].

**Figure 18:**
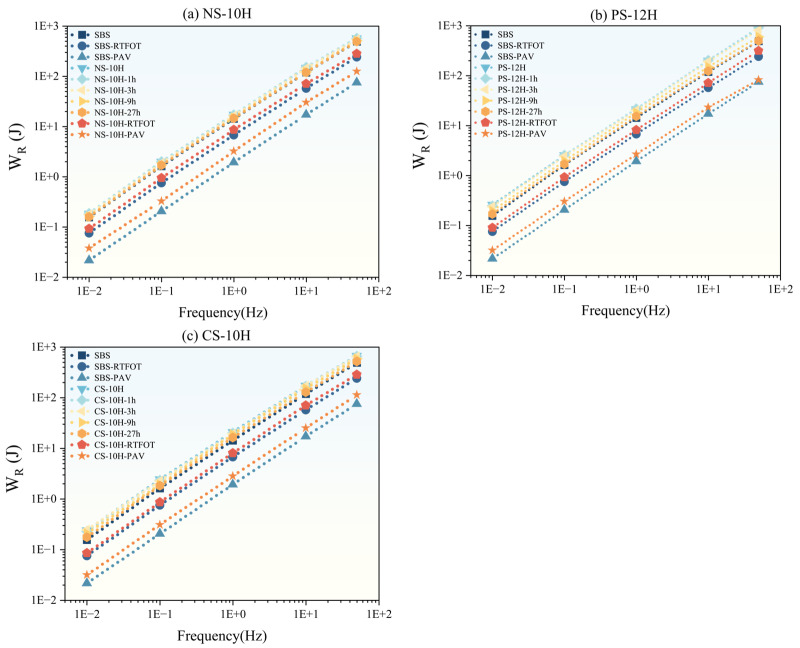
Evolution of W_R_ for different types of MFPA.

Using 70 °C as the reference temperature, the evolution of the high-temperature performance of MFPA was further investigated, as shown in Figure 18. Compared with unfoamed SBS-modified asphalt, the WR values of NS-10H, PS-12H, and CS-10H increased by 16%, 45%, and 36%, respectively; however, the enhancement gradually diminished over storage time, with approximately 50% of the improvement lost within the first hour after micro-foaming. After 3 h of storage, the foam structure completely dissipated, and the high-temperature performance of MFPA recovered to a level comparable to that of SBS-modified asphalt. Notably, after short-term and long-term aging, MFPA exhibited superior aging resistance, with its post-aging W_R_ values being 22% and 51% higher than those of SBS-modified asphalt, respectively. In conjunction with previous studies [11,12,13], these phenomena can be attributed to the following factors: First, the evaporation of crystallization water during the micro-foamed process absorbed heat, reducing the preparation temperature of polymer-modified asphalt and thereby mitigating thermo-oxidative aging during production. Second, the transient presence of microbubbles partially impeded the diffusion of oxygen into the asphalt matrix, thereby mitigating the aging process. Most importantly, as a physical modification technique, micro-foamed significantly improved the dispersion of SBS polymers within the asphalt, promoting the formation of a more complete and stable three-dimensional polymer network structure, which was largely preserved during aging.

### 3.8. Low-Temperature Cracking Resistance

**Figure 19:**
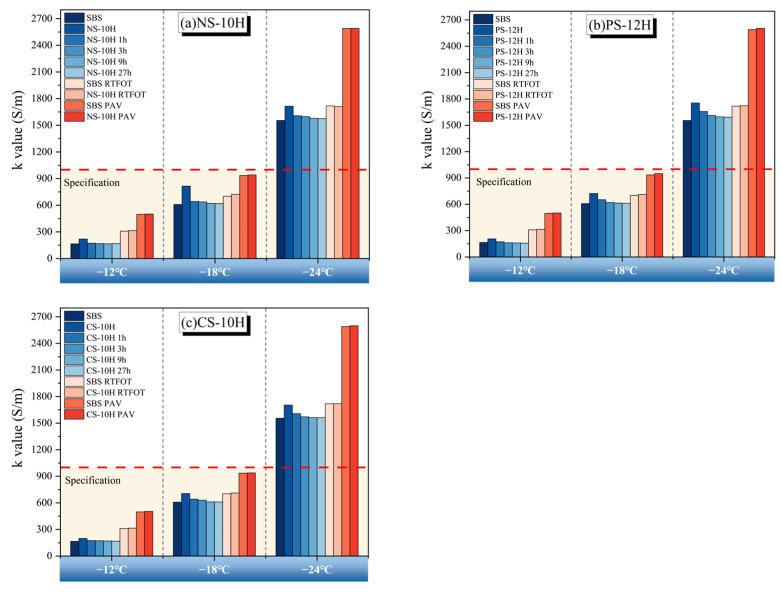
Evolution of low-temperature performance of MFPA.

Based on the ratio *k* value (S/m) obtained from the BBR test, the low-temperature performance of micro-foamed asphalt during the short-term foamed process and after different aging levels were evaluated. According to specifications, a *k* value below 1000 indicates acceptable low-temperature performance of asphalt. The test results are shown in Figure 19.

Overall, the micro-foaming process does not alter the fundamental behavior of SBS-modified asphalt at low temperatures, and the k value consistently increases with decreasing temperature. However, freshly prepared MFPA still contains residual moisture that cannot completely escape during the micro-foaming process. This residual water forms weakly bonded interface and pores within the asphalt matrix. Under low-temperature conditions, freezing-induced volumetric expansion generates internal stresses, leading to a temporary reduction in cracking resistance. Compared with unfoamed SBS-modified asphalt, the cracking resistance of NS-10H, CS-10H, and PS-12H decreased by approximately 24%, 17%, and 13%, respectively. With prolonged micro-foamed duration, the low-temperature performance gradually recovers. After 1 h of micro-foamed, the recovery ratios of cracking resistance for NS-10H, CS-10H, and PS-12H reached 79%, 64%, and 68%, respectively. Following 3 h of thermal storage, the low-temperature performance of MFPA was essentially restored to that of the original unfoamed SBS-modified asphalt. After RTFOT and PAV aging, the k-values of MFPA are comparable to those of SBS-modified asphalt, indicating that the micro-foamed process does not cause any pronounced deterioration in the low-temperature performance of MFPA after short-term or long-term aging. Compared with mechanical foamed methods reported in previous studies, the micro-foamed technique adopted in this study enables more complete moisture release, with no residual crystallization water retained in the polymer system; therefore, it does not induce permanent damage to the cracking resistance of asphalt during service.

Although the micro-foamed process slightly reduces the initial low-temperature cracking resistance due to the presence of transient residual moisture, this effect is temporary and rapidly diminishes during short-term thermal conditioning. From an engineering perspective, such a reduction is unlikely to impose significant restrictions on practical application, provided that MFPA is used after a brief holding period that allows sufficient moisture dissipation. In addition, the comparable k-values observed after RTFOT and PAV aging indicate that the micro-foamed process does not compromise long-term low-temperature durability. Therefore, MFPA is well-suited for typical pavement environments where construction temperatures are high enough to facilitate moisture release, while applications in extremely cold climates may require adequate conditioning time or moisture-monitoring procedures to ensure optimal performance.

### 3.9. Fatigue Modeling

Based on the VECD theory, the analysis of LAS test data can predict the fatigue life of asphalt under any load. Using Equations (10)~(17) in Section 2.3.9, the VECD model parameters for the various asphalt were obtained, as listed in Table 3.

**Figure 20:**
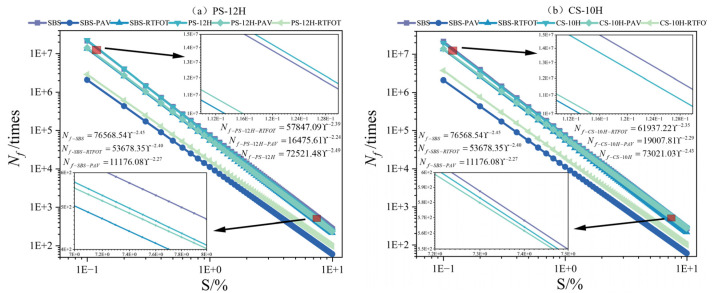

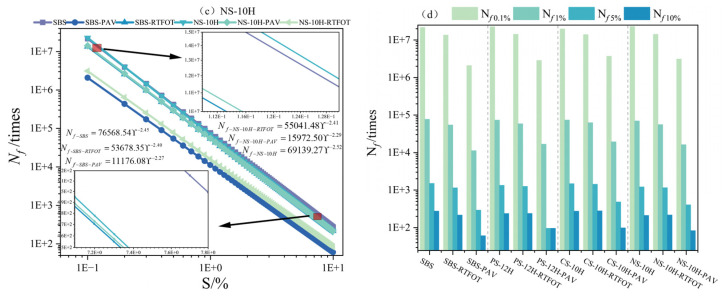
Low-temperature performance evolution of micro-foamed asphalt.

The asphalt fatigue life model constructed according to Equation (10) is presented in Figure 20a–c, while the predicted fatigue life at different strain levels (0.1%, 1%, 5%, and 10%) is shown in Figure 20d. Overall, the micro-foamed process does not alter the characteristic trends of fatigue performance of SBS-modified asphalt; with increasing strain levels, the fatigue life of all binders decreases markedly. Under 0.1% strain, the fatigue lives of SBS asphalt, NS-10H, PS-12H, and CS-10H were 2.16 × 10^7^, 2.29 × 10^7^, 2.24 × 10^7^ and 1.97 × 10^7^ loading cycles, respectively. Short-term and long-term aging reduced the fatigue performance of SBS-modified asphalt by 28% and 84%, respectively. Although the micro-foamed process has a limited impact on the fatigue performance of unaged and short-term aged SBS asphalt, after long-term aging, the fatigue lives of PS-12H, CS-10H, and NS-10H increased on average by 37%, 78%, and 49% compared to SBS asphalt, indicating that the micro-foamed treatment can significantly enhance the long-term fatigue resistance of asphalt systems. This phenomenon is primarily attributed to the fact that the fatigue resistance of SBS-modified asphalt largely relies on its polymer network structure, which undergoes irreversible degradation during long-term aging [12,49]. In contrast, MFPA interior develops a dense micro-foam structure that provides localized deformation buffering, thereby mitigating stress concentration and crack propagation. Furthermore, the micro-foamed process promotes a more uniform dispersion and improved compatibility of SBS within the asphalt, helping preserve the integrity of the polymer network during long-term aging and resulting in enhanced fatigue performance of MFPA under extended service conditions.

### 3.10. Morphological Evolution

As a mild and short-term reversible physical viscosity reduction approach, the viscosity reduction effect of micro-foamed process is significantly affected by the release of crystal water and the distribution of bubbles. Therefore, by investigating the attenuation and dissipation behavior of bubbles in MFPA, the viscosity reduction efficiency of the foamed process and the residual crystalline water content within the system can be quantitatively evaluated from the perspective of morphological evolution.

**Figure 21:**
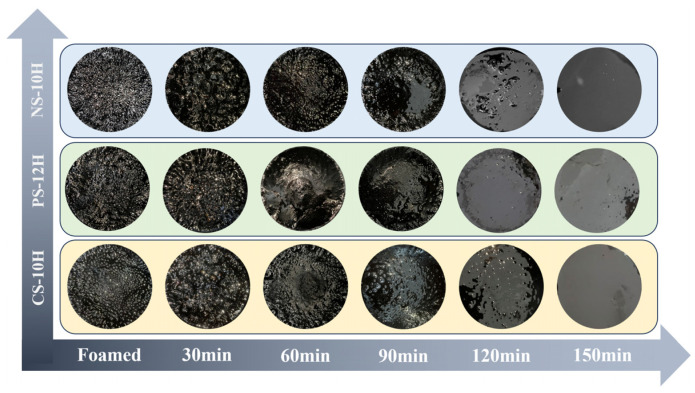
Morphological evolution of liquid apparent foam.

**Figure 22:**
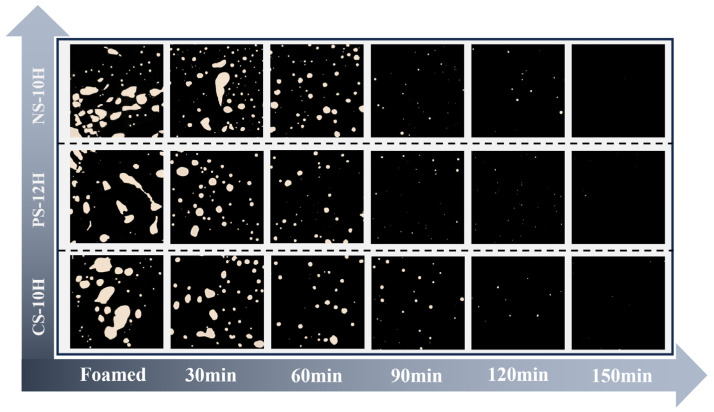
Morphological evolution of micro-bubble system (20×).

**Figure 23:**
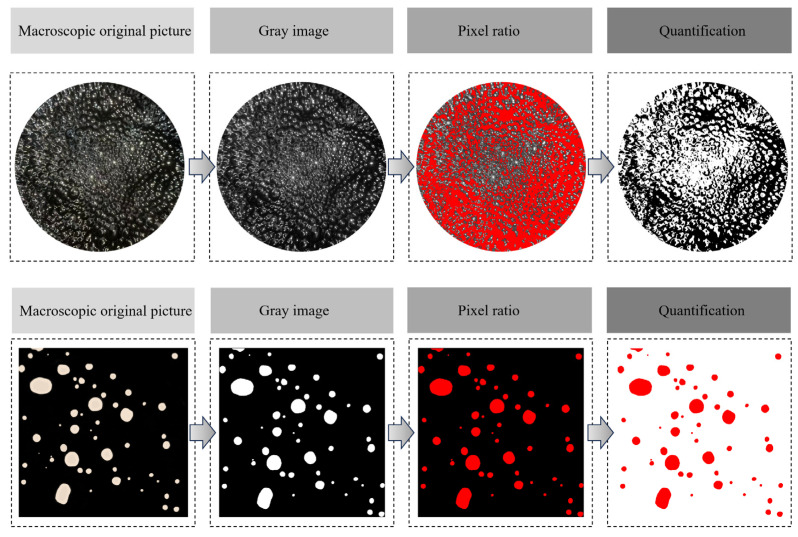
Image processing procedure.

The apparent foam morphology of MFPA is illustrated in Figure 21. During the initial stage of micro-foamed (≤60 min), the bubbles formed on the asphalt surface are relatively uniformly dispersed, and the system maintains a comparatively stable overall condition. As the release and evaporation of crystalline water gradually diminish, apparent bubbles begin to aggregate and coalesce, exhibiting a characteristic “island-like” distribution pattern. When the heat storage time is extended to 150 min, the apparent bubbles of MFPA almost completely dissipate, and the asphalt reverts to a smooth surface state comparable to that before foamed. From the microscopic scale, as shown in Figure 22, in the early stage of foamed (≤30 min), due to the sharp release of crystal water, the bubble size in the system is significantly different, and the average diameter is large. As the thermal storage time increases, both bubble density and average diameter decrease gradually, while the bubble morphology becomes more regular and approaches a near-circular geometry. During the period of 60–90 min, the foam system undergoes rapid collapse and attenuation. After 120 min, the release of crystalline water is essentially complete, and there is no obvious bubble residue within the system. Based on MATLAB, the macro and micro foam images are identified and the foam density of MFPA is quantified; the corresponding processing workflow is presented in Figure 23.

**Figure 24:**
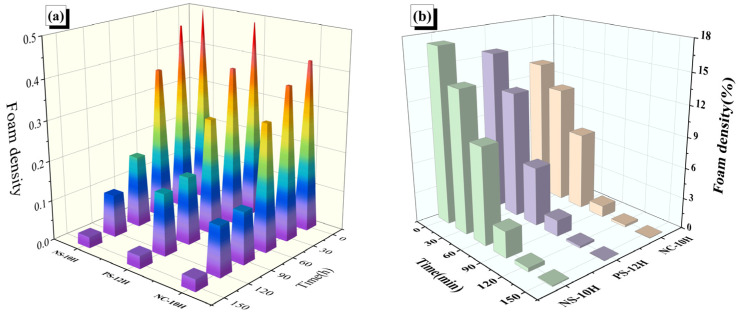
(**a**) Liquid apparent foam density; (**b**) System bubble density.

The quantified liquid apparent foam density and system foam density are shown in Figure 24. Within the first 60 min of the micro-foamed process, the liquid apparent and system foam densities of MFPA remain above 30% and 12%, respectively, maintaining a relatively stable state throughout this stage. The period between 60 and 90 min represents the most pronounced stage of foam attenuation. During this interval, the apparent foam densities of NS-10H, PS-12H, and CS-10H decreased by approximately 44%, 27%, and 50%, respectively, while their corresponding system foam densities declined by about 40%, 27%, and 48%. These comparable reduction trends indicate a good degree of consistency between apparent and system foam density in characterizing the foam dissipation rate. After 150 min of thermal storage, the release of crystalline water approaches completion, and both the apparent and system foam densities decrease to below 4% and 2%, respectively. Overall, under identical time conditions, the foam density ranking of the three asphalts follows the order: NS-10H > PS-12H > CS-10H. The macro-scale foam observed on the liquid surface represents the visible accumulation of micro-bubbles generated within the asphalt matrix. During the micro-foamed process, a portion of the internally formed bubbles migrate upward and coalesces at the surface, forming the apparent foam layer, while the remaining bubbles dispersed throughout the bulk phase constitute the system internal foam density. Accordingly, the attenuation of surface foam essentially reflects the dissipation of internal micro-bubbles, which explains the consistent reduction trends observed between apparent and system foam densities.

**Figure 25:**
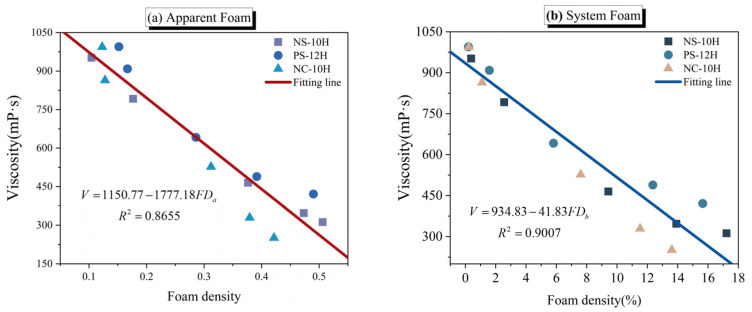
Correlation between macro- and micro-scale bubble densities and viscosity of MFPA.

In conjunction with the viscosity recovery curve test results presented in Section 3.3, a correlation analysis was conducted between apparent foam density, system internal foam density, and viscosity. As shown in Figure 25, the foam dissipation process of MFPA exhibits a strong linear correlation with viscosity recovery, with all correlation coefficient (R^2^) exceeding 0.85. Notably, the correlation between system internal foam density and viscosity is stronger than that observed for apparent foam density. This can be attributed to the fact that internal bubble characteristics are directly governed by the release of crystalline water, thereby exerting a more direct influence on viscosity reduction. In contrast, apparent bubbles primarily originate from vapor rising and accumulating at the liquid surface; their formation is accompanied by gas evaporation and variations in surface tension [24,54], both of which may interfere with the accuracy of their representation of viscosity reduction performance. Nevertheless, owing to its ease of rapid observation, apparent foam density retains practical value and can serve as an auxiliary indicator for the preliminary evaluation and prediction of viscosity reduction performance at specific stages of MFPA.

## 4. Conclusions

This study investigated the effects of a micro-foaming process based on crystalline hydrates with high water of crystallization on the viscosity reduction efficiency, physicochemical properties, compatibility, and adhesion performance of SBS-modified asphalt. Furthermore, the high- and low-temperature rheological behavior, fatigue life, and pore structure evolution of MFPA under short-term and long-term service conditions were comprehensively evaluated. The main conclusions are summarized as follows:

(1) When the dosages of NS-10H, CS-10H, and PS-12H were 5%, 3%, and 3%, respectively, MFPA exhibited optimal foamed performance, with *ER_max_* reaching 8–10 and *HL* remaining stable at approximately 180 s. Regarding FI, peak values of 1086.37, 1164.94, and 1228.70, respectively, were attained. The construction temperature of the mixture was reduced by an average of 10–15%, and the viscosity reduction effect remained stable within 60 min after foaming.
(2) The compaction and mixing temperatures of MFPA exhibit a sigmoidal functional relationship with *ERmax*, while the dt value derived from the viscosity recovery curve shows a linear correlation with *HL*. Therefore, *ERmax* and *HL* can be employed to rapidly evaluate the construction temperature and viscosity recovery degree of MFPA. Moreover, the bubble dissipation process demonstrates a pronounced linear correlation with viscosity recovery of the system, indicating that foam density can serve as an auxiliary indicator for the preliminary evaluation and prediction of viscosity reduction performance at MFPA-specific stages.
(3) The micro-foamed process represents a short-term physical viscosity reduction approach that does not introduce new functional groups into the system. Compared with SBS-modified asphalt, MFPA exhibits a 6% increase in softening point, a 12% decrease in ductility, and no significant change in penetration. The micro-foamed behavior facilitates polymer dispersion and enhances system fluidity, thereby improving compatibility; the CV and SE values increase by approximately 55% and 76%, respectively.
(4) The micro-foamed process generates dense pores within the SBS network, forming an internal supporting structure that facilitates the absorption of cyclic loading stresses and provides elastic buffering. However, the retention of trace moisture may induce internal stresses at low temperatures. Compared with unfoamed SBS asphalt, MFPA exhibits a 32% improvement in rutting resistance and a 6% increase in VECD fatigue life.
(5) While reducing construction temperature, the micro-foamed process improves polymer dispersion and compatibility within the system and enhances workability, thereby contributing to improved aging resistance of the asphalt. After RTFOT and PAV aging, MFPA demonstrates 22% and 51% enhancements in rutting resistance, respectively, and 5% and 55% improvements in fatigue performance compared with unfoamed SBS-modified asphalt, whereas the low-temperature performance remains essentially unchanged.

Future work should further clarify the long-term pore evolution and its interaction with environmental aging to ensure the durability of micro-foamed SBS asphalt. In addition, establishing predictive relationships between foaming parameters, viscosity recovery, and construction temperature would support more accurate process control and broader engineering applications.

## Figures and Tables

**Table 1:** Physical properties of AH-90 matrix asphalt.

Technical Index	Specification	Test Result	Test Method
Penetration (25 °C, 0.1 mm)	80~100	79.9	ASTM D5 [30]
Softening point (T&B, °C)	≥43	46.1	ASTM D36 [31]
Ductility (5 °C, cm)		8.97	ASTM D113 [32]
Viscosity (135 °C, mPa⋅s)		399.2	ASTM D4402 [33]

**Table 2:** Properties of SBS 792.

Property	Parameter	Method
Structure	Radial	
Styrene-butadiene ratio	40/60	
Tensile strength (MPa)	≥24	ASTM D412 [34]
Breaking elongation (%)	≥730	ASTM D412
Tensile stress at 300% (%)	≥3.5	ASTM D412

**Table 3:** VECD model parameters.

Project	$C_{1}$	$C_{2}$	α	$D_{f}$	k
SBS	0.0228	0.6125	1.4095	33.2987	1.3875
SBS-PAV	0.0272	0.5833	1.2827	36.3339	1.4167
SBS-RTFOT	0.12387	0.5098	1.1802	40.3158	1.4902
PS-12H	0.02223	0.6001	1.3891	31.254	1.3999
PS-12H-PAV	0.02675	0.5715	1.2956	35.1852	1.4285
PS-12H-RTFOT	0.08185	0.5138	1.1494	39.8505	1.4862
CS-10H	0.02322	0.5879	1.3743	32.4398	1.4121
CS-10H-PAV	0.02193	0.5655	1.2486	33.5605	1.4345
CS-10H-RTFOT	0.06671	0.4894	1.1394	34.9321	1.5106
NS-10H	0.02669	0.5983	1.4261	33.2447	1.4017
NS-10H-PAV	0.0342	0.5491	1.3106	37.9529	1.4509
NS-10H-RTFOT	0.08765	0.5057	1.1684	38.9918	1.4943

## Data Availability

Data is contained within the article.
